# Effect of Neutron Irradiation on the Electronic and Optical Properties of AlGaAs/InGaAs-Based Quantum Well Structures

**DOI:** 10.3390/ma16206750

**Published:** 2023-10-18

**Authors:** Aleksey N. Klochkov, Almas Yskakov, Aleksander N. Vinichenko, Danil A. Safonov, Nikolay I. Kargin, Maksim V. Bulavin, Aleksey V. Galushko, Vladik R. Yamurzin, Ivan S. Vasil’evskii

**Affiliations:** 1 MEPhI Institute of Nanoengineering in Electronics, Spintronics and Photonics, Moscow 115409, Russiasafonov.dan@mail.ru (D.A.S.); ivasilevskii@mail.ru (I.S.V.); 2 Joint Institute for Nuclear Research, Dubna 141980, Russia; almas.9@mail.ru (A.Y.); vampeer33@gmail.com (V.R.Y.); 3 Faculty of Physics and Technics, L.N. Gumilev Eurasian National University, Astana 010008, Kazakhstan; 4 Institute of Nuclear Physics, Almaty 050032, Kazakhstan

**Keywords:** InGaAs, two-dimensional electron gas, neutron irradiation, photoluminescence, electron concentration, mobility, high resolution X-ray diffraction

## Abstract

The effect of neutron irradiation on the structural, optical, and electronic properties of doped strained heterostructures with AlGaAs/InGaAs/GaAs and AlGaAs/InGaAs/AlGaAs quantum wells was experimentally studied. Heterostructures with a two-dimensional electron gas of different layer constructions were subjected to neutron irradiation in the reactor channel with the fluence range of 2 × 10^14^ cm^−2^ ÷ 1.2 × 10^16^ cm^−2^. The low-temperature photoluminescence spectra, electron concentration and mobility, and high-resolution X-ray diffraction curves were measured after the deactivation. The paper discusses the effect of neutron dose on the conductivity and optical spectra of structures based on InGaAs quantum wells depending on the doping level. The limiting dose of neutron irradiation was also estimated for the successful utilization of AlGaAs/InGaAs/GaAs and AlGaAs/InGaAs/AlGaAs heterostructures in electronic applications.

## 1. Introduction

High-quality heterostructures based on the InGaAs active layer have high electron mobility and are widely used in microwave heterostructure electronics, infrared lasers, and detectors, photovoltaic cells, and sensors. Many electronic devices operate in harsh environments, including ionizing radiation. Low-noise amplifiers based on pseudomorphic high electron mobility transistor (PHEMT) heterostructures with the InGaAs channel are used in communication systems, including satellite communications and space missions. The study of the radiation resistance of heterostructure electronics is also necessary to create sensors and other control devices that will be used for a long time in the facilities with very high neutron fluences (more than 10^18^ cm^−2^); for example, in nuclear installations, accelerators, and thermonuclear reactors [1]. There is a high demand for low magnetic field Hall sensor devices with low power consumption that can be made into III-V semiconductors such as AlGaAs/InGaAs/GaAs heterostructures [2,3,4]. These have attracted increasing interest recently by virtue of their high electron mobility combined with moderate sheet carrier densities, low temperature dependence of the output Hall voltage, and large signal-to-noise ratios. Magnetic field sensors based on InAs structures have good long-term sensitivity stability under neutron irradiation up to a fluence of 10^17^ cm^−2^ [1,5].

Neutron irradiation of InGaAs-based semiconductor heterostructures and devices was shown to result in different degradation effects [6,7]. Irradiation with fast neutrons with fluences up to 10^14^ cm^−2^ led to a significant decrease in the light power generated via the InGaAs p-i-n photodiode and to photodiode dark current degradation due to the formation of lattice defects [8]. Neutron irradiation leads to a decrease in the output power of solar cells based on InGaAs due to a decrease in the lifetime of minority charge carriers scattered in traps due to displacement damage [9]. The InGaAs single heterojunction bipolar transistors irradiated up to a 6 × 10^14^ cm^−2^ 1 MeV equivalent neutron fluence, showing significant current gain degradation [10]. Neutron irradiation of AlGaAs/GaAs HEMTs up to fluences of 5 × 10^14^ neutrons·cm^−2^ has shown that their variations in static, small-signal, and noise parameters undergo rather small changes [11]. At large, fast neutron fluences of 10^16^ cm^−2^, the vanishing of the two dimensional electron gas was observed in GaAs/AlGaAs HEMTs [12]. The most significant contribution to the degradation of GaAs/AlGaAs HEMTs and to the decrease in the 2-DEG density is attributed to the deep traps introduced via neutron irradiation in the AlGaAs donor layer [13]. The fast neutron (1 MeV) irradiation damage was shown to result in the decrease in the drain current of AlGaAs/GaAs and InGaP/InGaAs HEMTs and the damage coefficient of AlGaAs HEMTs is about one order greater than that of InGaAs HEMTs for the same radiation source [14]. The InGaAs-containing pseudomorphic HEMTs were shown to have better ionizing radiation tolerance as compared to GaAs MESFETs and GaAs/AlGaAs HEMTs [15]. In most studies, radiation testing has typically focused on electronic devices rather than basic semiconductor materials. However, it is difficult to utilize the data on radiation-stimulated changes in device parameters (saturation and leakage currents, gain factors, breakdown voltage) to extract information about the mechanisms of ionizing radiation action on devices. Therefore, studies on the resistance of fundamental optical, electrical, and structural properties of semiconductor heterostructures on ionizing neutron irradiation are essential.

Semiconductor nanoheterostructures based on the InGaAs narrow-gap active layer with GaAs or AlGaAs claddings are usually doped with n- or p- type dopants through a thin spacer layer separating the channel from the doping region to create high conductivity. For electronic applications, donor doping is a standard, and electrons move in a narrow-gap InGaAs layer, with minimal scattering by impurity ions. The characteristic thicknesses of the active layers of nanoheterostructures range from 1 to 100 nm. Therefore, the processes of defect formation and radiation modification of such structures might differ significantly from those inherent to the bulk semiconductor materials.

Within the scope of current research endeavors, calculations of reaction rates and the classification of secondary elements and particles are actively conducted utilizing the Geant4 software environment and Jendl cross-section libraries [16,17,18,19]. These refined analytical methods enable us to delve deeper into the impact of the neutron flux on semiconductors, refining the dynamics of reactions under various conditions. Special attention is directed towards elucidating the influence of both fast and thermal neutrons on the processes governing defect formation and the generation of free charge carriers.

In this work, we experimentally studied the effect of neutron irradiation on the optical and electronic properties of doped heterostructures with AlGaAs/InGaAs/GaAs and AlGaAs/InGaAs/AlGaAs quantum wells (QWs) and different electron concentrations. After bombardment by neutrons, defects and various scattering centers are formed in heterostructures, the concentration of which can be judged from the change in the electron mobility, especially at low measurement temperatures [20]. Also, the formation of defects in the semiconductor heterostructures of the AlGaAs/GaAs/AlGaAs and AlGaAs/InGaAs/GaAs types can be indirectly studied by the shape and intensity of their photoluminescence spectra. Early studies of the degradation of optical properties at high neutron fluxes concerned mainly bulk GaAs crystals. It was noticed that new transitions appeared in the low-temperature photoluminescence spectra at the bandgap edge for the fluence ~10^16^ cm^−2^, due to the formation of high density defect states [21,22,23,24]. The photoconductivity spectra taken on the fast-neutron-irradiated samples of GaAs show the appearance of optical transitions between the deep energy levels and either the valence or the conduction band lying at approximately 0.2 and 0.7 eV above the valence band and 0.5 eV below the conduction band [25]. Partial recovery of optical properties is possible via high-temperature vacuum annealing at T = 550 °C [21]. It has been shown that a neutron fluence of ~10^14^ cm-2 leads to an increase in the threshold current of a GaAs/AlGaAs laser diode without noticeable changes in the spectral parameters [26]. Thus, the change in the optical properties of bulk GaAs under neutron irradiation is well understood, but that of heterostructures with quantum wells is a poorly studied area.

The purpose of this work is to study the dose effects during the neutron irradiation of epitaxial semiconductor nanoheterostructures with a quantum well based In_x_Ga_1-x_As (x ~ 0.21)’s electronic and structural properties in the fluence range of 2 × 10^14^ cm^−2^ ÷ 1.2 × 10^16^ cm^−2^ to assess and analyze the radiation resistance of modern materials for the electronic component base of microwave and sensor electronics based on arsenide heterostructures.

## 2. Materials and Methods

**Figure 1:**
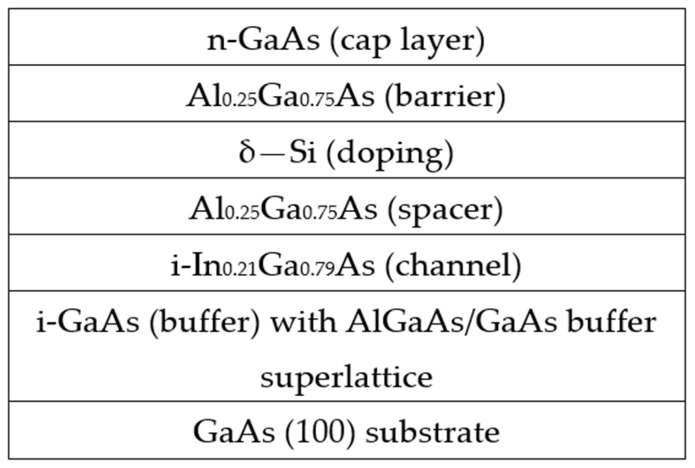
Layer structure of samples with Al_0.25_Ga_0.75_As/In_0.21_Ga_0.79_As/GaAs quantum well and one-sided δ-doping.

The samples under study were grown via molecular beam epitaxy (MBE) on a Riber Compact 21-T facility at MEPhI on semi-insulating GaAs substrates with a (100) crystallographic orientation. Two types of nanoheterostructures were studied, differing in the composition of the quantum well cladding and the Si donor doping strategy. Figure 1 shows a diagram of the Al_0.25_Ga_0.75_As/In_0.21_Ga_0.79_As/GaAs QW samples with one-side delta doping. The thickness of the In_0.21_Ga_0.79_As QW layer is 10.5 nm. The doping delta layer of silicon atoms is located relative to the QW closer to the surface of the structure through the undoped Al_0.25_Ga_0.75_As spacer. The three single-sided doped heterostructures were studied (#435, #468, and #485), differing by the concentration of silicon atoms in the doping layer and in the spacer thickness and resulting in the different electron densities in the structures. The samples’ surface was protected by a thin GaAs cap layer. An I-GaAs buffer layer was grown between the QW and the substrate to smooth the growth surface, which additionally contained a short-period GaAs/AlGaAs superlattice at the initial stage of growth. The total buffer thickness was more than 500 nm.

The second type of heterostructures studied (sample #328) contained an Al_0.25_Ga_0.75_As/In_0.21_Ga_0.79_As/Al_0.25_Ga_0.75_As quantum well, which was doped by Si δ-layers on both sides. A feature of sample #328, in contrast to the layered scheme shown in Figure 1, was the presence of a lower 50 nm Al_0.25_Ga_0.75_As barrier layer, in which an additional δ doping layer was located, as separated from the QW by an AlGaAs spacer. The lower δ-layer had a three times smaller Si concentration than in the upper δ-layer. Bilateral doping provided a significant increase in the electron concentration in the QW of heterostructure #328 in comparison to the single-doped samples.

**Figure 2:**
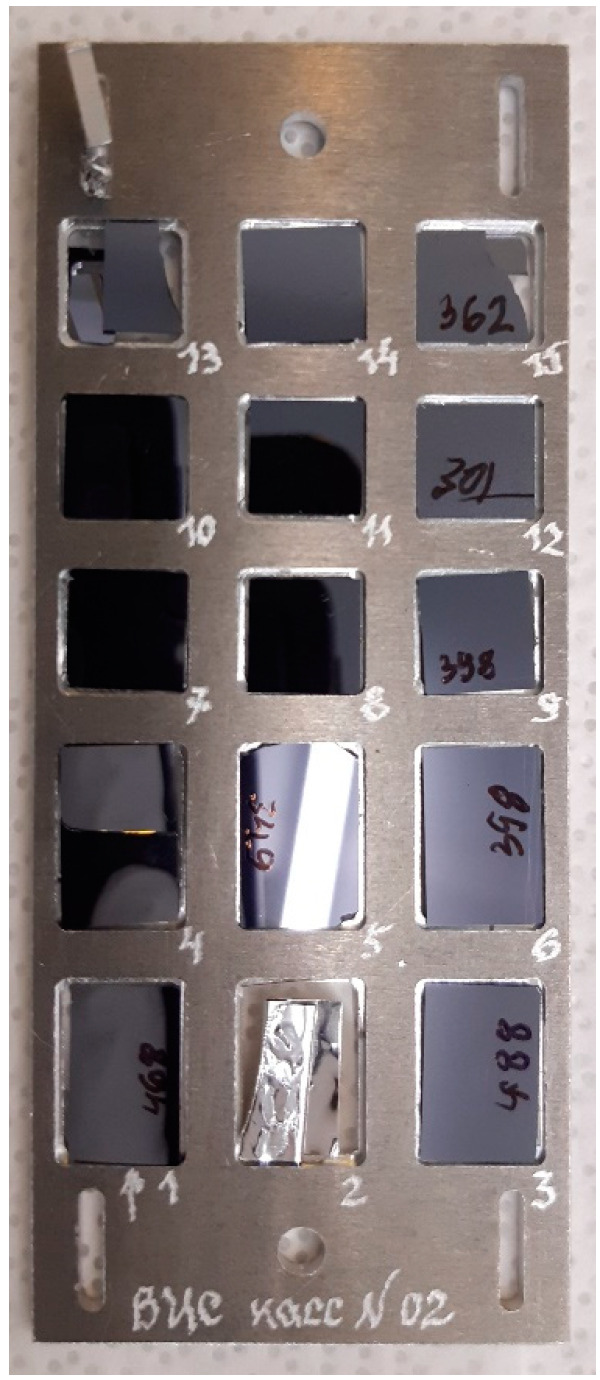
Photo of a holder cassette with several heterostructures.

Irradiation of materials via the full spectrum of reactor neutrons was carried out on channel No. 3 of the IBR-2 pulsed nuclear reactor (JINR, Dubna, Russia) with an average power of 1.6 MW. The neutron spectrum is continuous in the range from several meV (thermal neutrons) to fast neutrons with energies up to 9 MeV. Detailed features of the channel were published elsewhere [27]. To study the effect of the neutron irradiation dose on the electrical, optical, and structural properties of heterostructures based on InGaAs, substrates with the heterostructures under study were scribed and divided into rectangular pieces with an area of about 1 cm^2^. Then, the pieces of heterostructures were fixed on three different holder cassettes (Figure 2). The holder was made of a plate (aluminum alloy) with rectangular recesses to accommodate the samples and protect their surface from the scratches and kicks by the thin aluminum screen plates. During installation in channel No. 3 of the IBR-2 reactor, different holder cassettes were located simultaneously but at the different distances from the reaction zone. The neutron radiation dose is the same for the samples on one cassette and is determined via the distance from the cassette to the surface of water moderator.

The samples were irradiated with different neutron fluences of 2 × 10^14^ n/cm^2^, 3.2 × 10^15^ n/cm^2^, and 1.2 × 10^16^ n/cm^2^. These fluences will be denoted by doses No. 1, 2, and 3, respectively.

The low-temperature photoluminescence (PL) spectra of the samples were measured at the temperature T = 77 K. A solid-state green laser with a wavelength of 532 nm was used as pumping. The luminescence signal was collected along the normal direction to the sample surface and guided to the input slit of the Horiba iHR-550 spectrometer via quartz fiber. The spectra were detected via the cooled silicon CCD matrix. The samples of the same series were measured in a single cycle with the same adjustment of the system and laser intensity for the correct intensity comparison. The photon counting time was varied depending on the samples luminescence brightness in order to match the photodetector optimal dynamic range and was subsequently scaled during the intensity calculation.

The concentration and mobility of the two-dimensional electron gas at 77 K and 300 K were determined via the four-probe van der Pau technique by measuring the Hall effect and the electrical resistance on an Ecopia HMS-5000 setup. The clover-leaf Hall bar mesa was prepared using standard photolithography and wet etching. The samples were subjected to rapid thermal annealing at 380 °C for 3 min to reduce the resistance of applied ohmic contact metallization. Since AlGaAs/InGaAs structures have a fairly high Schottky barrier, electron transport measurements require the annealing of the ohmic contacts after metallization. Typical Ni/Ge/Au ohmic metallization materials are strongly activated by neutron irradiation, which makes it difficult to study the electrical properties of the samples after neutron irradiation due to an increase in the deactivation period, as well as the changes in the resistance of metal ohmic contacts. Thus, rapid thermal annealing was a necessary step for the ex situ measurement strategy. Early studies of thermal annealing of neutron-irradiated semiconductors indicate that typical annealing temperatures exceed 400 °C, which has a sufficient effect on the deep-trap structural defects in neutron-irradiated GaAs [28,29]. The thermal annealing could lead to the partial recovery of radiation-induced property degradation [14]. Thus, the influence of the annealing procedure on the measured electrical properties of the material under study cannot be ruled out, but the temperature and time of the high-temperature annealing treatment were selected to minimize this influence. Fast thermal annealing was performed only for the samples subjected to electron transport measurements. The optical spectra were measured on the samples before annealing.

High-resolution X-ray diffraction measurements were carried out using a Rigaku Ultima IV diffractometer with X-ray beams from a Cu Kα1 copper tube (U = 30 kV, I = 30 mA, and λ = 1.5406 Å). The measurements were performed using a Ge (220) 2-bounce monochromator with an angular step of 0.002° and a counting time of 5 s.

## 3. Results

### 3.1. Electrophysical Properties

Table 1 shows the concentration and mobility of the two-dimensional electron gas in the studied heterostructures as a function of the neutron fluence. The measurements reveal some differences in the electrical characteristic behaviors of the single-side doped QW samples (#485) and double-side doped QW (#328), depending on the neutron fluence. When analyzing the data, note that the heterostructures pieces for the different fluences were taken from different parts of the wafer and therefore had a natural spread of initial parameters due to wafer area inhomogeneity within the value of 3%.

For sample 485-0, we observe a standard behavior for a degenerate two-dimensional electron gas in an Al_0.25_Ga_0.75_As/In_0.21_Ga_0.79_As/GaAs QW, in which the sheet electron concentration n_S_ is practically temperature independent due to the complete dopants’ ionization in the AlGaAs barrier. After neutron irradiation of the #485 heterostructure with doses 1 and 2, the electron concentration remained nearly unchanged. We observe only a slight decrease in n_S_ at 300 K for sample 485-2. However, electron mobility in QW of heterostructure 485 decreased after the first exposure to neutron radiation. The most significant decrease in mobility is observed at the measurement temperature of 77 K. After the third dose of neutron irradiation, the resistance of sample 485-3 was too high to reliably measure the concentration and mobility of charge carriers in our setup. An assessment of the mobility in sample 485-3 was made by taking a measurement at 77 K after illuminating the sample.

The electron concentration n_S_ for the as-grown double-side doped sample 328-0, in contrast to sample 485-0 depends significantly on the measurement temperature. The decrease in n_S_ as the temperature is lowered from 300 K to 77 K indicates the carriers freezing out due to the decrease in the thermal ionization of Si dopants in two δ-layers in the AlGaAs barriers. This might be due to the high doping when the Si dopant energy becomes close to the Fermi level, resulting in the incomplete ionization of impurities. Additionally some of the dopants have DX-like deep levels due to the high enough Al content in the AlGaAs-doped layer. In contrast to sample 485-1, after neutron dose 1 for sample 328-1, we observe a prominent decrease in both the electron mobility and concentration. An increase in the neutron fluence in sample 328-2 results in a further decrease in the two-dimensional electron density at the room temperature measurements, as well as a decrease in the electron mobility. Note in this case, for sample 328-2, the electron concentration temperature dependence practically disappears as compared to sample 328-0. Apparently, after irradiation in the reactor with a second neutron dose, shallow acceptor defects are formed in the heterostructures and the Fermi level becomes lower at the QW area. These compensating centers lead to more complete Si donor ionization. As a result, the scattering in the quantum well, which we observe in samples 328-1 and 328-2, is enhanced, and the difference in electron concentrations at the two measurement temperatures decreases. In sample 328-3, we observed a substantial conductivity decrease, which excludes the further use of heterostructures of this type after the neutron exposure with a fluence of the order of 1.2 × 10^16^ n/cm^2^. However, the conductivity is still measurable in sample 328-3 as compared to sample 485-3 due to the higher concentration before irradiation.

For samples #328 and #485, electron mobility differs before irradiation due to the different layer designs and dopant distribution profiles. For the second neutron fluence, the mobility converges for the samples of both types, especially at low temperatures. This indicates the emergence of a new dominant mechanism of electron scattering, which arose in both structures as a result of irradiation with a neutron flux.

**Figure 3:**
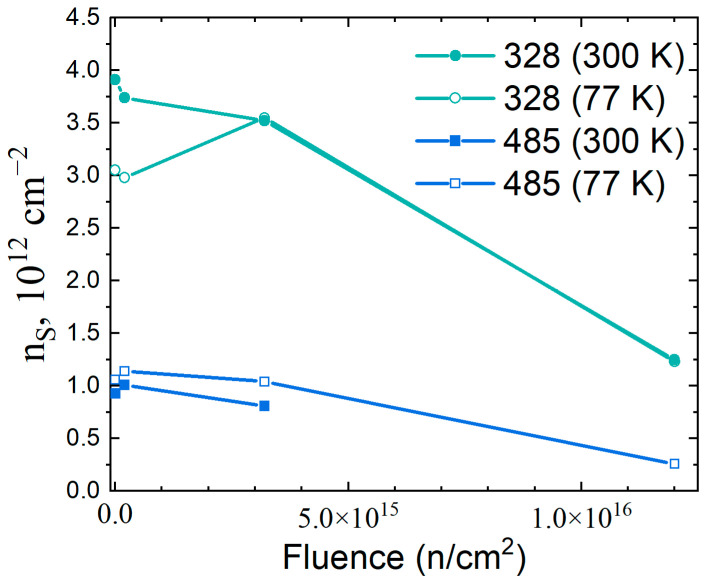
Dependence of the sheet electron concentration in InGaAs-based quantum wells on neutron fluence.

Figure 3 shows the dependence of the sheet electron concentration on the neutron fluence. The carrier removal rate coefficient, indicating the ratio of the electron concentration change to the cumulative neutron fluence, can be estimated from Table 1. Based on the room temperature data, the rate of carrier removal at the transition from dose 2 × 10^14^ cm^−2^ to 3.2 × 10^15^ cm^−2^ is approximately the same for samples 485 and 328 and is 7 × 10^10^ cm^−2^ electrons per 10^15^ cm^−2^ neutrons. However, the rate of the carrier removal is nonlinear with fluence and increases after the third neutron fluence to 2.5 × 10^11^ cm^−2^ electrons per 10^15^ cm^−2^ neutrons. Thus, the dependence of the conductivity of a two-dimensional electron gas in a QW on the neutron fluence has a threshold character. The electron concentration is almost unaffected by the neutrons at a fluence less than or equal to 3.2 × 10^15^ cm^−2^, and the electron mobility decreases monotonically with the increasing dose. After the exposure to the neutrons with a fluence of 1.2 × 10^16^ cm^−2^, a sharp increase in the resistance of the samples and a decrease in the electron mobility in the InGaAs QW were observed.

### 3.2. Photoluminescence Spectroscopy

**Figure 4:**
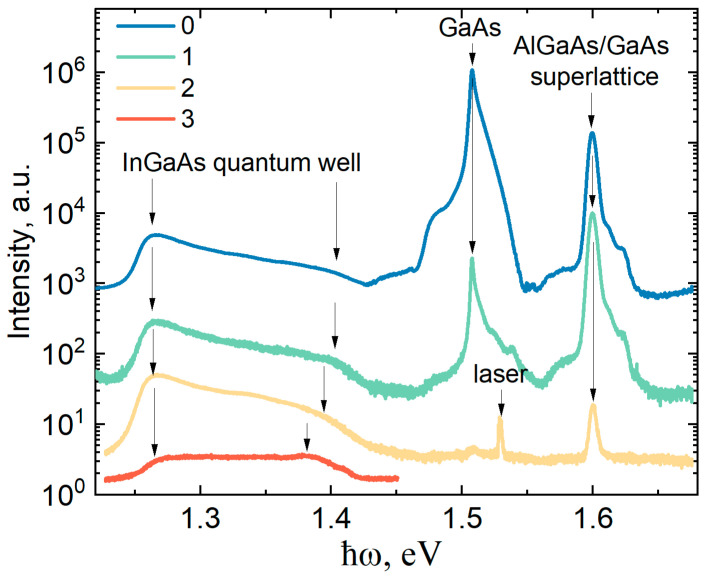
PL spectra at 77 K of heterostructures with double-side doped Al_0.25_Ga_0.75_As/In_0.21_Ga_0.79_As/Al_0.25_Ga_0.75_As QWs (sample 328) after exposure to various neutron fluences.

Let us consider the influence of the neutron radiation dose on the PL spectra of heterostructures with QWs. Figure 4 shows a series of the PL spectra of double-side doped Al_0.25_Ga_0.75_As/In_0.21_Ga_0.79_As/Al_0.25_Ga_0.75_As QWs (sample #328). In the PL spectrum of sample 328-0, we observe several luminescence bands originating from different heterostructure areas as it well known from the preliminary studies [30]: the emission from the quantum well in the range of 1.24–1.42 eV, the interband luminescence line from the GaAs buffer layer (1.508 eV) accompanied by the impurity band shoulder at 1.48 eV, as well as the sharp intensive peak from a GaAs/Al_0.25_Ga_0.75_As buffer short-period superlattice with a maximum at 1.6 eV.

Neutron irradiation significantly reduces the PL intensity integrally, while different lines in the spectrum showed the different intensity depressions upon the neutron irradiation. Thus, in sample 328-0, the most intense luminescence line comes from a thick GaAs buffer layer at a photon energy of 1.508 eV. After irradiation with a dose of 1, the PL amplitude at the GaAs interband recombination maximum becomes weaker than the GaAs/AlGaAs superlattice peak. After a neutron dose of 3, only weak luminescence from InGaAs QW remains in the PL spectrum.

The PL spectrum from InGaAs QW with a high electron concentration has a wide linewidth due to the wide energy spectrum of the degenerate electron gas. Electrons occupy quantum states with energies ranging from the edge of the first size-quantization subband E_1_ to the Fermi level E_F_, which can be more than 100 meV higher than the energy E_1_ at a sufficiently high doping level. The width of the PL spectrum from a QW with 2DEG is often proportional to the sheet electron concentration [31,32,33].

After the exposure to neutron radiation, the shape of the PL spectrum from InGaAs QWs for samples 328-1 and 328-2 is similar to the spectrum of sample 328-0. The position of the peaks and the width of the luminescence spectrum of QW-related transitions do not change with the decreasing signal intensity. However, after the exposure to dose 3 of neutron radiation, we observe a change in the shape and a decrease in the width of the PL spectrum from InGaAs QWs. This is in good agreement with the electrophysical measurements (Table 1), according to which the sheet electron concentration in samples 328-0, 328-1, and 328-2 is practically the same at T = 77 K, while n_S_ decreases significantly in sample 328-3.

It follows from Figure 4 that the luminescence spectra from the different layers of the nanoheterostructure have different intensity sensitivities to the neutron fluence. After the exposure to neutron radiation, the luminescence is most rapidly quenched from the bulk undoped GaAs semiconductor layers, in which nonradiative recombination centers are formed under the action of ionizing neutron radiation. Quantum heterostructures such as GaAs/AlGaAs superlattices trap electrons and holes in quantum-localized states, resulting in a decrease in carrier diffusion and an increase in the radiative recombination probability. The intensity of photoluminescence from a doped quantum well with a degenerate electron gas is least affected by neutron radiation. In this case, the QW already contains free electrons, the concentration of which weakly depends on the neutron fluence (except for dose 3). Therefore, the PL intensity in this case is mainly determined by the number of holes diffusing to the quantum well, which is determined via the nonradiative hole lifetime and depends on the trap concentration. In the case of interband recombination in the GaAs layers and in the AlGaAs/GaAs superlattices, the PL intensity is determined both via the nonradiative lifetime of holes and electrons.

**Figure 5:**
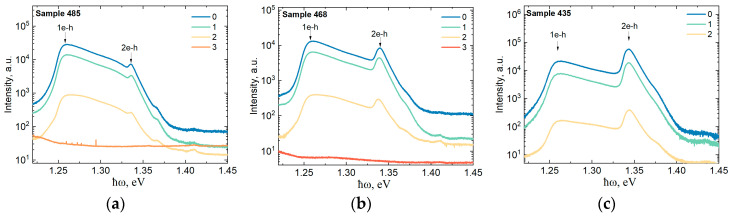
PL spectra at 77 K of single-sided doped heterostructures with Al_0.25_Ga_0.75_As/In_0.21_Ga_0.79_As/GaAs QWs after exposure to various neutron fluences: (**a**) sample 485; (**b**) sample 468; and (**c**) sample 435.

The photoluminescence spectra of the samples of heterostructures with one-side doped Al_0.25_Ga_0.75_As/In_0.21_Ga_0.79_As/GaAs QWs are shown in Figure 5. Samples 485, 468, and 435 differed in the concentration of silicon atoms in the δ layer and the thickness of the Al_0.25_Ga_0.75_As spacer layer. This led to different concentrations of two-dimensional electrons in the QW, which are listed in Table 2.

In contrast to the double-side doped heterostructure (sample 328), in the case of single-side doped structures, we observe only broad luminescence bands in the photon energy range of 1.22–1.4 eV (Figure 5). We explain the absence of recombination lines from the GaAs buffer layer and the AlGaAs/GaAs superlattices by the fact that in samples 435, 468, and 485, there is no lower AlGaAs wide-band barrier layer between the buffer and the InGaAs QW (Figure 1). As a result, free electrons and holes that are produced in the GaAs buffer layer upon photoexcitation in the course of the PL measurement easily transfer to a narrower-gap InGaAs QW layer.

In the spectra of samples 435-0, 468-0, and 485-0, two PL peaks are observed, which are indicated in Figure 5 as 1e-h and 2e-h and correspond to the interband recombination involving the first- and second-size quantization electronic subbands in the QW, respectively. With an increase in the layer electron concentration in the QW, we observe an increase in the relative intensity of the PL peak with the participation of the second size-quantization subband 2e-h (Figure 5a–c), as well as its width. This corresponds to an increase in the filling of the second subband with electrons.

After the samples of the Al_0.25_Ga_0.75_As/In_0.21_Ga_0.79_As/GaAs heterostructures are irradiated with neutrons, the photoluminescence intensity significantly decreases, and after dose 3, we could not observe the spectral features of the samples. The shape of the PL spectra remains practically unchanged after the neutron exposure: the positions of the peaks in a series of the samples with different doses differ by no more than 1 meV. This pattern is in good agreement with the results of the measurements of the PL spectra of sample 328 (Figure 4), as well as with the results of the measurements of the neutron dose dependence of the electron concentration in the QW (Table 1). According to these measurements, the electron concentration in the InGaAs QW is independent of the neutron fluence up to a dose of 2.

**Figure 6:**
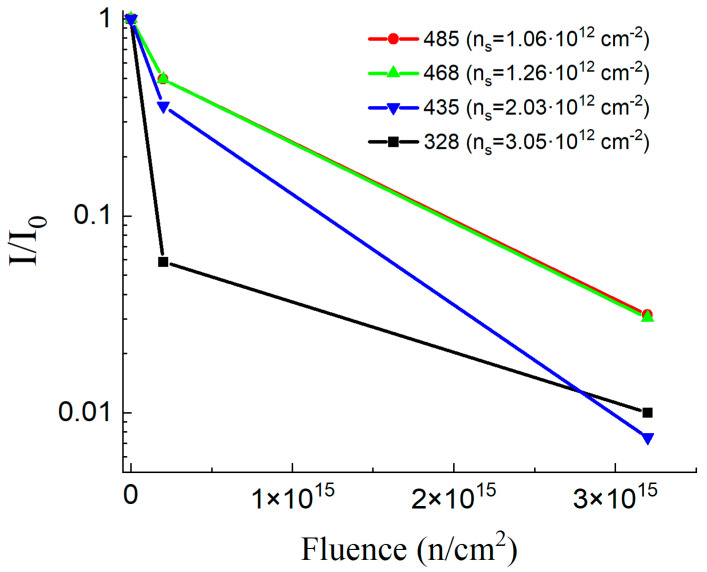
PL intensity suppression of QW optical transition depending on the neutron fluence.

The PL intensity from the InGaAs layer in the studied samples depends differently on the neutron fluence. Figure 6 plots the attenuation coefficient of the intensity I of the radiative transition 1e-h in four studied samples depending on the dose of neutron radiation with respect to the initial intensity I_0_. Note that for a small neutron fluence, the intensity suppression is more prominent, and for a 1e15 fluence, the suppression rate slows down. We observe similar dependences of the attenuation coefficient I/I_0_ for samples 468 and 485 with a lower electron density n_S_. In samples 435 and 328, a much stronger decrease in the PL intensity is observed after doses 1 and 2 of neutron radiation. Since the construction of all the studied heterostructures is almost identical (with the exception of sample 328), we assume that the features observed in Figure 6 are associated with different levels of doping of the studied heterostructures. For the same reason that led to a stronger mobility sensitivity to the neutron fluence in the heavily doped 328 heterostructure compared to 485 (Table 1), excess neutral donor impurities in the vicinity of the QW can affect the scattering rates and the intensity of charge carrier recombination.

### 3.3. High Resolution X-ray Diffractometry

X-ray diffraction (XRD) analysis is one of the precision techniques for monitoring the composition of heterostructure layers, their thickness, and the degree of crystalline ordering. In this work, the method of double-crystal high-resolution diffractometry was used to evaluate the effect of neutron irradiation on the crystal structure of multilayer AlGaAs/InGaAs/GaAs heterostructures. With an increase in the density of structural defects or a noticeable smearing of heterointerfaces, the X-ray diffraction peaks are expected to broaden, as well as the suppression of intensity and thickness oscillations.

**Figure 7:**
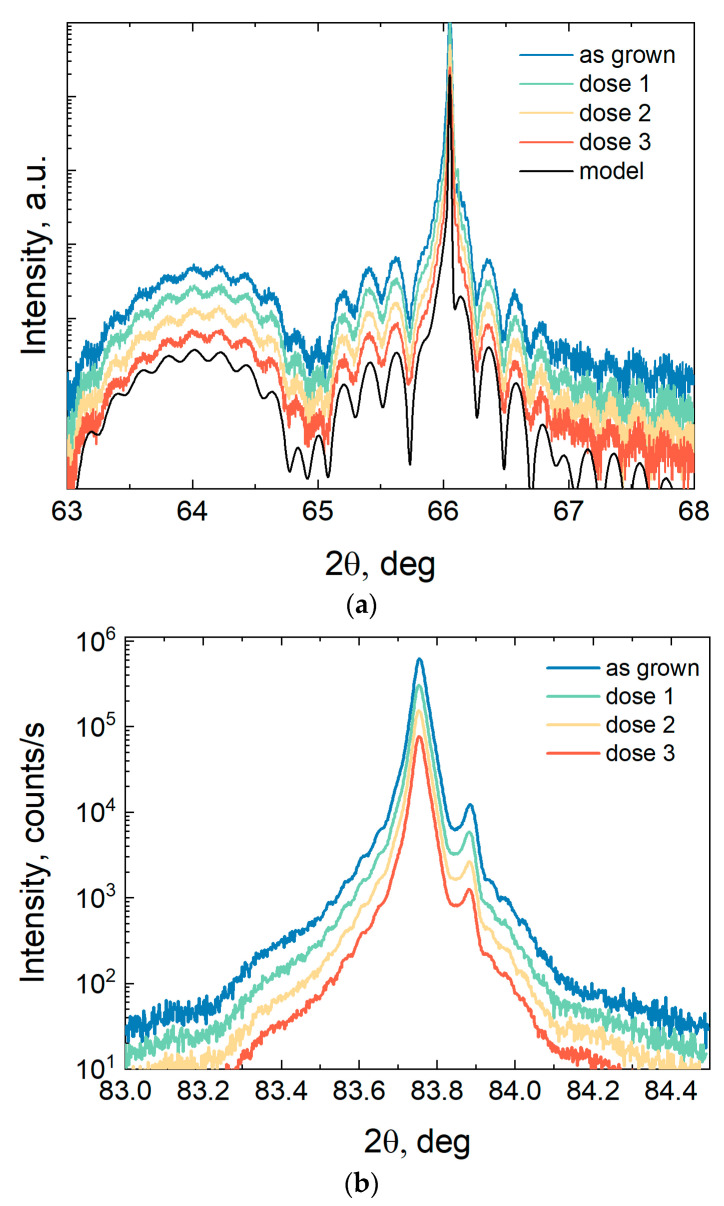
Diffraction reflection curves in 2θ-ω geometry for a heterostructure with Al_0.25_Ga_0.75_As/In_0.21_Ga_0.79_As/GaAs QW before and after irradiation with three doses of neutrons (plots are shifted vertically for clarity): (**a**) symmetrical reflection from planes (004), (**b**) asymmetric reflection (422) for a grazing incident beam.

Figure 7 shows the measured diffraction and reflection curves in the 2θ-ω geometry of a one-side doped heterostructure Al_0.25_Ga_0.75_As/In_0.21_Ga_0.79_As/GaAs (samples #468) in the symmetric reflection (004) and grazing incidence asymmetric reflection (422). The symmetric reflection curve (Figure 7a) exhibits the following features: a sharp intense peak from the GaAs substrate at 2θ = 66.05°, a broad peak from the InGaAs layer with a large lattice parameter relative to GaAs at 64°, and intensity oscillations, the period of which is determined via the total thickness of the upper layers InGaAs and AlGaAs of heterostructures. Similar features are observed in the reflection curves of the asymmetric reflection (422) (Figure 7b). The intensity profiles were calculated in the Rigaku GlobalFit program for a model heterostructure, which made it possible to determine the parameters of the In_x_Ga_1-x_As layer via the profile fitting method. The content of indium in the In_x_Ga_1-x_As layer is x = 0.215 ± 0.003, and the layer thickness is 10.4 ± 0.5 nm in accordance with the values specified during MBE growth.

Figure 7 shows that, for heterostructure 468, the shape of the X-ray diffraction curves does not change after neutron irradiation. As the neutron fluence increases, neither the amplitude nor the width of the various peaks on the XRD curves change for the studied fluence range. We also do not observe a change in the width of the ω-rocking curves of the GaAs (004) peak (figure not shown). Thus, for the studied range of neutron fluences, there is no noticeable degradation of the X-ray diffraction fine structure for AlGaAs/InGaAs/GaAs quantum-well heterostructures. There is no evident change neither in the lattice parameters of the InGaAs and AlGaAs nanolayers (otherwise it would lead to a shift in the maxima angular positions), nor in the heterointerface broadening or noticeable appearance of cluster defects (otherwise it would lead to an increase in the half-width of the peaks and an increase in the background intensity far from the main peaks). The weak dependence of the crystal structure of AlGaAs/InGaAs/GaAs nanoheterostructures on the neutron irradiation dose is similar to the properties of bulk GaAs layers [34].

## 4. Conclusions

Thus, in this work, the effect of neutron radiation on the optical and electronic properties of doped heterostructures with AlGaAs/InGaAs/GaAs and AlGaAs/InGaAs/AlGaAs quantum wells was experimentally studied. It is found that the dependence of the conductivity of a two-dimensional electron gas in a QW on the neutron fluence has a threshold character. Thus, the electron concentration is almost unaffected by neutrons at a fluence less than or equal to 3.2 × 10^15^ cm^−2^, and the electron mobility decreases monotonically with the increasing dose. After the exposure to neutrons with a fluence of 1.2 · 10^16^ cm^−2^, a sharp increase in the resistance of the samples and a decrease in the electron mobility in the InGaAs QW were observed. The intensity of the low-temperature photoluminescence spectra decays with an increase in the dose of neutron radiation due to the formation of nonradiative recombination centers in the heterostructures. The luminescence spectra from different layers of the heterostructures have different intensity sensitivities to the neutron fluence. After the exposure to neutron radiation, the luminescence is most rapidly quenched from the bulk undoped GaAs semiconductor layers and the undoped GaAs/AlGaAs superlattices. The intensity of photoluminescence from a doped quantum well with a degenerate electron gas is least affected by neutron radiation. It follows from the results of the work that the limiting neutron radiation fluence for the successful operation of this type of AlGaAs/InGaAs/GaAs and AlGaAs/InGaAs/AlGaAs heterostructures on GaAs substrates is approximately 3 × 10^15^ cm^−2^. A further increase in the neutron dose leads to the catastrophic degradation of the heterostructure’s electronic properties.

## Figures and Tables

**Table 1:** Sheet concentration n_s_ and mobility µ of electrons in Al_0.25_Ga_0.75_As/In_0.21_Ga_0.79_As/Al_0.25_Ga_0.75_As (sample #328) and Al_0.25_Ga_0.75_As/In_0.21_Ga_0.79_As/GaAs quantum well heterostructures (sample #485) at room temperature (T = 300 K) and liquid nitrogen boiling point (T = 77 K) after different doses of neutron radiation.

Sample		Neutron Fluence, cm^−2^	T = 77 K		T = 300 K	
			n_s_, 10^12^ cm^−2^	µ, cm^2^/V·s	n_s_, 10^12^ cm^−2^	µ, cm^2^/V·s
#485	0	-	1.06	26,220	0.93	7210
	1	2 × 10^14^	1.14	26,400	1.01	7190
	2	3.2 × 10^15^	1.04	12,780	0.81	4880
	3	1.2 × 10^16^	0.26 *	250 *	-	-
#328	0	-	3.05	33,180	3.91	7080
	1	2 × 10^14^	2.98	23,420	3.74	6810
	2	3.2 × 10^15^	3.55	12,950	3.48	5720
	3	1.2 × 10^16^	1.23	1190	1.25	1070

* After illumination.

**Table 2:** Sheet concentration n_s_ and mobility µ of electrons in investigated Al_0.25_Ga_0.75_As/In_0.21_Ga_0.79_As/GaAs heterostructures.

Sample	n, 10^12^ cm^−2^	μ, cm^2^/V·S	n, 10^12^ cm^−2^	μ, cm^2^/V·s
	300 K		77 K	
435	2.03	7500	1.88	29,300
468	1.26	7100	1.26	22,000
485	0.93	7210	1.06	26,220

## Data Availability

Not applicable.
